# Soluble Variants of Human Recombinant Glutaminyl Cyclase

**DOI:** 10.1371/journal.pone.0071657

**Published:** 2013-08-15

**Authors:** Cristiana Castaldo, Silvia Ciambellotti, Raquel de Pablo-Latorre, Daniela Lalli, Valentina Porcari, Paola Turano

**Affiliations:** 1 Pharmacology Department, Siena Biotech, Siena, Italy; 2 Magnetic Resonance Center (CERM), University of Florence, Sesto Fiorentino, Florence, Italy; 3 Department of Chemistry, University of Florence, Sesto Fiorentino, Florence, Italy; Oak Ridge National Laboratory, United States of America

## Abstract

Recombinant human Glutaminyl Cyclase expressed in *E. coli* is produced as inclusion bodies. Lack of glycosylation is the main origin of its accumulation in insoluble aggregates. Mutation of single isolated hydrophobic amino acids into negative amino acids was not able to circumvent inclusion bodies formation. On the contrary, substitution with carboxyl-terminal residues of two or three aromatic residues belonging to extended hydrophobic patches on the protein surface provided soluble but still active forms of the protein. These mutants could be expressed in isotopically enriched forms for NMR studies and the maximal attainable concentration was sufficient for the acquisition of ^1^H-^15^N HSQC spectra that represent the starting point for future drug development projects targeting Alzheimer’s disease.

## Introduction

Glutaminyl-peptide Cyclotransferase (QPCT), also known as Glutaminyl Cyclase (QC), catalyzes the conversion of N-terminal L-glutaminyl peptide residues to pyroglutamyl groups, a process required for the maturation of numerous bioactive peptides [1]. QPCTs are widespread and have been isolated from animals, plants, and bacteria [2].

**Figure 1:**
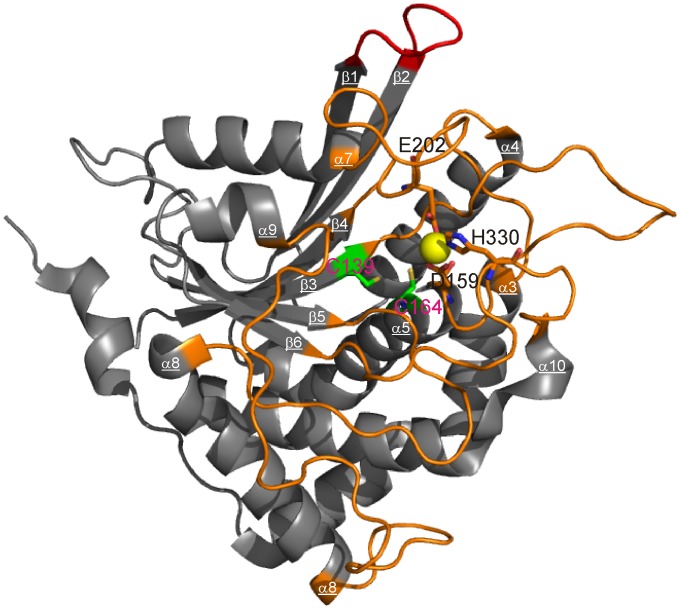
3D structure of hQPCT. Ribbon representation of the X-ray structure of hQPCT (PDB id 2AFM). The zinc ion is shown by a yellow sphere, the zinc ligands are shown as orange sticks and the two Cys residues responsible for the disulphide bridge formation as green sticks. The loop connecting β1 with β2 is highlighted in red, while those forming the crown-like structure around the zinc are in orange.

Mammalian QPCTs are zinc-dependent glycoproteins [3]. The structure of the human enzyme (hQPCT) exhibits a typical α/β-hydrolase fold whose characteristic features are a central six-stranded β-sheet surrounded by α-helices [4] that is common to other mammalian proteins [5] (Fig. 1). Recombinant forms obtained in *E. coli* are non-glycosylated and therefore much less soluble [6]. Murine and human proteins recombinantly expressed in the yeast *Pichia pastoris* are instead glycosylated and their X-ray structure has revealed some loop rearrangements in the neighborhood of the active center [5], the extent of these rearrangements being smaller for hQPCT.

Human QPCTs are considered potential candidates in the formation of pGlu-modified amyloid peptides in Alzheimer’s disease (AD) and their inhibition attenuates AD-like symptoms in mice [7].

Given this role, QPCT is an important target for drug development in AD. The possibility to use the well-established solution NMR approaches to screen libraries of potential QPCT inhibitors depends upon the ability to express soluble protein forms with different isotope enrichment schemes. NMR approaches for drug screening are based on the chemical shift perturbation mapping of the protein residues measured in ^1^H-^15^N HSQC spectra of ^15^N-enriched protein upon addition of the ligand. The mapping is possible whenever the following conditions are met: *i*) a structure of the protein and *ii*) the assignment of the ^1^H-^15^N HSQC spectrum are available [8], [9], [10], [11]. While the first condition has been achieved by X-ray crystal structure determinations (PDB id: 2ZED, 3SI1, 2AFM, 3PBB), [4], [5], [12], [13] no NMR assignment exists yet for hQPCT, nor ^1^H-^15^N HSQC spectra have been reported. Given the size of the protein, this might be accomplished via triple resonance NMR experiments that require ^15^N,^13^C and partial ^2^H enrichment on protein forms with a solubility of hundreds of µM [14]. To this purpose, we have designed mutations aimed at increasing protein solubility for its expression in *E. coli* [15]. Mutations sites were identified from an analysis of the hydrophobic solvent exposed surface areas. Single point mutations of isolated hydrophobic residues, namely F260E, L289E and I47E, were not sufficient for our purposes. These residues are located in different areas far from the active site. The observed behaviour suggested that the low solubility of hQPCT is not due to a generic hydrophobic character of the protein surface but rather to some specific interactions that may give rise to self-aggregation. The best candidate surfaces for intermolecular self-recognition were identified as two extended hydrophobic patches located in relative proximity from the active site. A hypothesis-driven modelling of the formation of aggregates involving these hydrophobic regions suggested that there are three candidate residues belonging to these hydrophobic areas but far enough from the zinc centre to guarantee unaffected enzymatic activity upon mutation. They are Y115, Y117 on one hydrophobic area and W149 on the other. The double mutant Y115E-Y117E (2xmut, hereafter) indeed resulted in a large increase in protein solubility that allowed expression of the protein in its soluble and active form and recording of good signal/noise HSQC spectra that may be useful for drug screening projects. A 6xmut, designed for purposes outside the scope of our paper, but containing the three key mutations Y115E-Y117E-W149D was found to be almost equally soluble and active.

## Results and Discussion

### Expression and Purification of hQPCT

Different expression vectors and hosts have been used to produce the recombinant human glutaminyl cyclase [2], [3], [16]. For example, Huang and collaborators [16] have tested two different bacterial vectors (pET32 and pET43.1) containing different tags (Thioredoxin-tag, Nus-tag, S-tag, His_6_-tag) with the intention of improving protein solubility [5]. In order to simplify the purification protocol and minimize the loss of protein due to multiple purification steps, we have cloned the hQPCT cDNA in an expression vector (pQE80L) containing a single His_6_-tag at the N-terminus of the protein. Several expression trials have been performed in order to test different *E. coli* strains (BL21DE3, Origami B), growth media (richer SuperBroth or minimal M9 media), incubation temperatures (17°C, 21°C, 25°C and 37°C), incubation times (24 and 48 h) and IPTG concentrations (0.2, 0.5 and 1 mM). Differently to what reported for the expression of hQPCT in pET vectors [5], from our expression trials the best condition turned out to be 17°C, 0.2 mM IPTG for 48 hours in rich medium using BL21DE3 as *E. coli* strain (see Materials and Methods).

**Figure 2:**
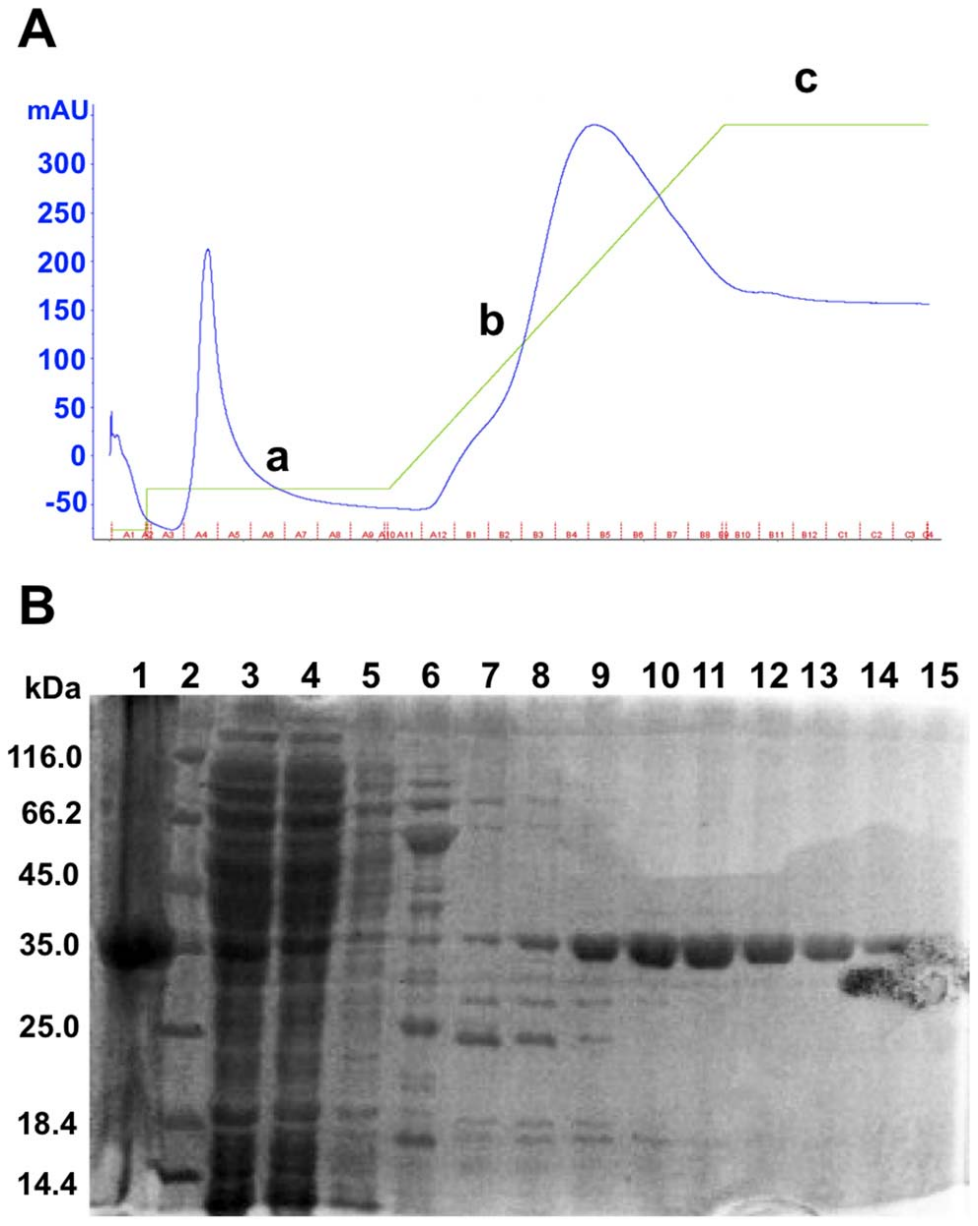
Purification of wild type hQPCT. **A,** Imidazole gradient (green line) (a) 50 mM, (b) 50–500 mM, (c) 500 mM in FPLC Akta (GE Healthcare). Blue line: UV measure (mAU). **B,** SDS-PAGE of purified protein fractions. Lane 1: insoluble fraction, lane 2: protein marker, lane 3: total fraction, lane 4: flow-through, lane 5: wash unbound, lanes 6–7: fractions 50 mM imidazole, lanes 8–15: fractions 50–500 mM imidazole.

After the first step of purification (Ni-affinity column), we checked the purity by loading fractions on an acrylamide gel (Fig. 2). Fractions obtained with an imidazole gradient correspond to the His_6_-tagged hQPCT (Fig. 2A). However, almost 80% of protein was found in inclusion bodies (lane 1) whereas only a 20% of protein was recovered in the soluble fraction (lanes 8–14) (Fig. 2B). These fractions were then pooled and the protein concentration measured. The final yield of hQPCT after the first step of purification was around 30 mg/l. However, most of the protein aggregated as demonstrated by the analytical size exclusion chromatography (Superdex 5/150 column) performed on a small volume of sample (50 µl). No further size exclusion in HiLoad 16/60 Superdex 75 column was applied.

An aliquot of purified hQPCT was demetalated for mass analysis by MALDI. The mass of the apo-hQPCT was 38735 Da, as expected on the basis of the protein sequence.

The circular dichroism spectroscopy (CD) analysis performed on hQPCT indicated a dominant α-helix content in the overall secondary structure of the protein, consistent with what has been reported in literature, where the calculation of the secondary structure elements revealed an α-helix and β-sheet content of 47% and 16% respectively [6], and coherent with the X-ray structure of hQPCT that reports 36% of α-helices and 16% of β-sheets [4].

**Figure 3:**
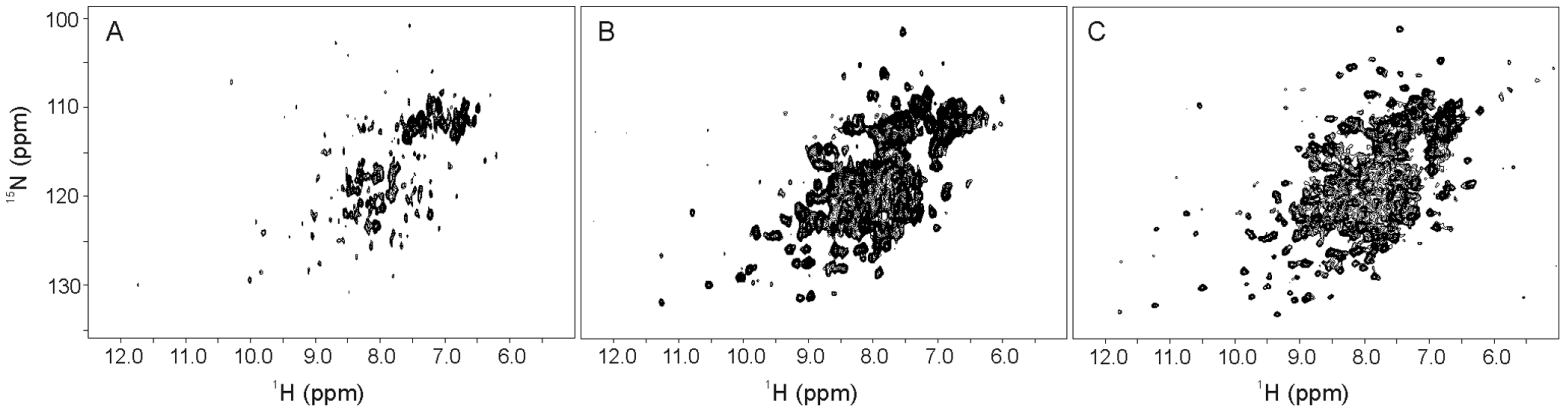
NMR spectra of wild type, 2xmut and 6xmut. 2D ^1^H-^15^N-HSQC spectra of: **A,** 30 µM wild type hQPCT at 700 MHz; **B,** of 90 µM 2xmut hQPCT at 950 MHz; **C,** 70 µM 6xmut hQPCT at 950 MHz. Spectra were recorded at 298 K, in 150 mM NaCl and 50 mM Tris pH 8 buffer.

The HSQC spectrum of hQPCT at its highest achievable concentration (30 µM) was acquired (Fig. 3A). Unfortunately the protein was not stable in solution and prone to precipitation. By addition of positive-charged amino acids in the protein solution (50 mM L-arginine) [17], it was possible to achieve a higher concentration of hQPCT (50 µM). Unfortunately, the effect was only temporary and the protein started to precipitate after 2 hours.

### Design of Active Multiple Mutants of hQPCT (2xmut hQPCT and 6xmut hQPCT)

**Figure 4:**
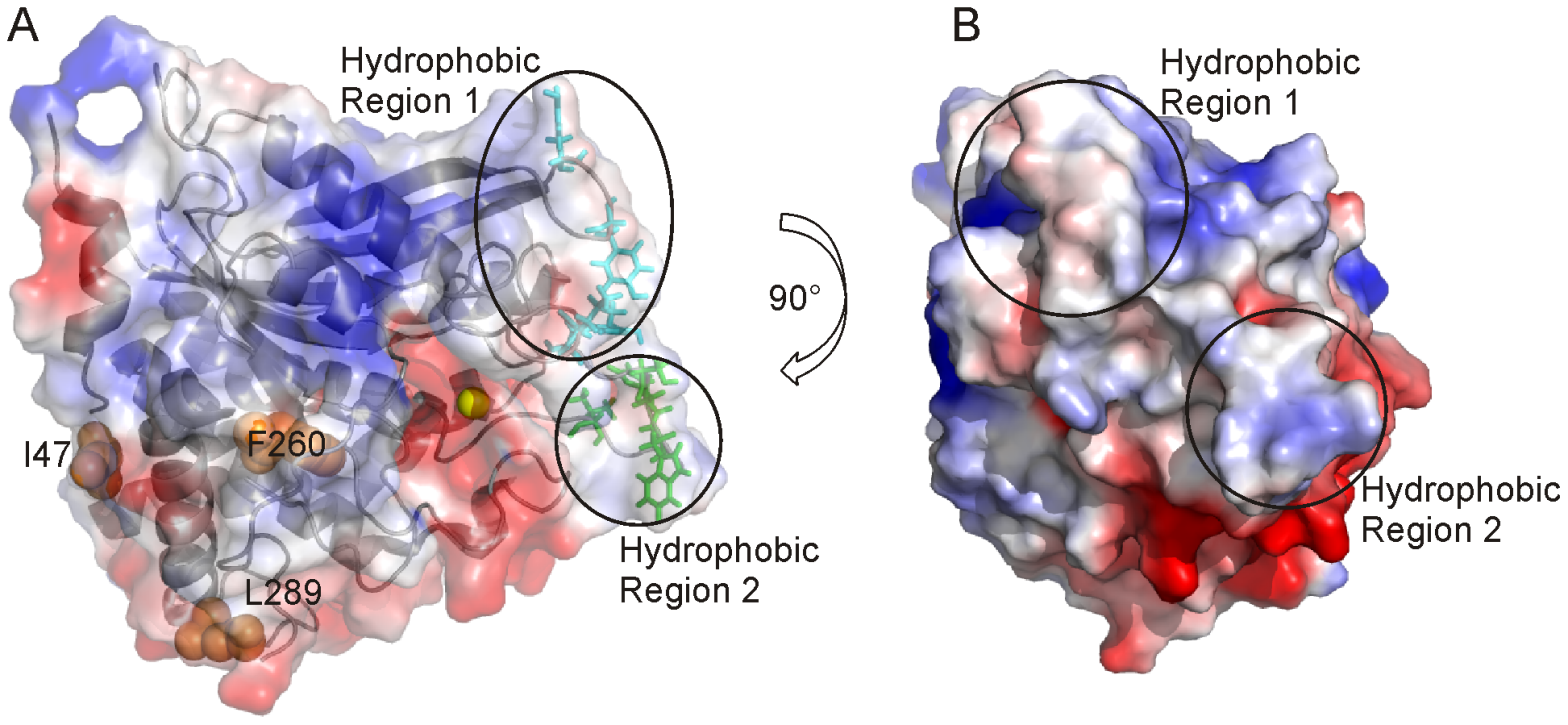
Surface charge representation of hQPCT, single mutants and residues used as input for docking calculation. **A,** Surface charge representation of hQPCT (PDB id 2AFM) where region of positive, negative and neutral electrostatic potential are indicated in blue, red and white, respectively. The electrostatic surfaces were generated using the software PyMOL with the command “generate vacuum electrostatic”. The protein orientation is the same as in Fig. 1. The ribbon representation of the protein is visible in transparency. Amino acids belonging to the hydrophobic regions 1 and 2 used as input for docking calculation are clustered into two groups and shown as cyan and green sticks, respectively. Single point mutations I47, L289, F260 are highlighted as orange spheres. The zinc ion is represented as a yellow sphere. **B,** Different views of hQPCT that allow visualizing the location of the hydrophobic regions 1 and 2 on the protein surface.

In order to increase hQPCT solubility, single point mutations were designed. Usually, the introduction of negatively charged residues in place of hydrophobic ones increases solubility and reduces protein aggregation in the same way as glycosylation does [18], [19], [20]. In hQPCT, hydrophobic solvent exposed residues, situated on loops or at the end of α-helices to avoid destabilization of the secondary structure, and far away from the active site, to avoid effects on the catalytic efficiency, were selected for directed-mutagenesis into hydrophilic residues: namely F260E, L289E and I47E (Fig. 4A).

At variance with previous observations in other large enzymes [21], [22], these single mutations were not sufficient to increase the solubility of hQPCT that was still mainly expressed in inclusion bodies. For this reason, it was necessary to design multiple mutants. Our working hypothesis was that the low solubility was due not to a generic hydrophobicity of the protein surface, but it’s rather related to the possibility to establish intermolecular contacts that can induce oligomerization. To simulate the formation of protein dimers, a structural model of the hQPCT homodimer was obtained with the program HADDOCK [23]. From the analysis of the electrostatic surface generated by PyMOL (The PyMOL Molecular Graphics System, Version 1.5.0.4 Schrödinger, LLC) together with the evaluation of the atomic accessible surface performed by Naccess [24] (see Materials and Methods), two main hydrophobic solvent exposed patches close to the active site and probably responsible for the hQPCT aggregation, were identified (Fig. 4A and 4B ). The candidate residues for mutations must be far enough from the active site in order to keep unchanged the activity of the enzyme. Residues less than 11 Å apart from active site (144, 146, 160, 201, 207, 248, 304, 305, 319, 325, and 329) are reported to decrease or even block the enzymatic activity [4], [25], therefore, they were not taken into account for possible mutations. All the solvent exposed residues belonging to the hydrophobic regions mentioned above, and located at more than 11 Å from the zinc ion, were defined as active residues (namely, 115, 117, 145, and 205 belonging to the hydrophobic region 1; 147, 148, 149 and 153 belonging to the hydrophobic region 2) and used as input for the docking calculation, as described in Materials and Methods (Fig. 4A).

**Figure 5:**
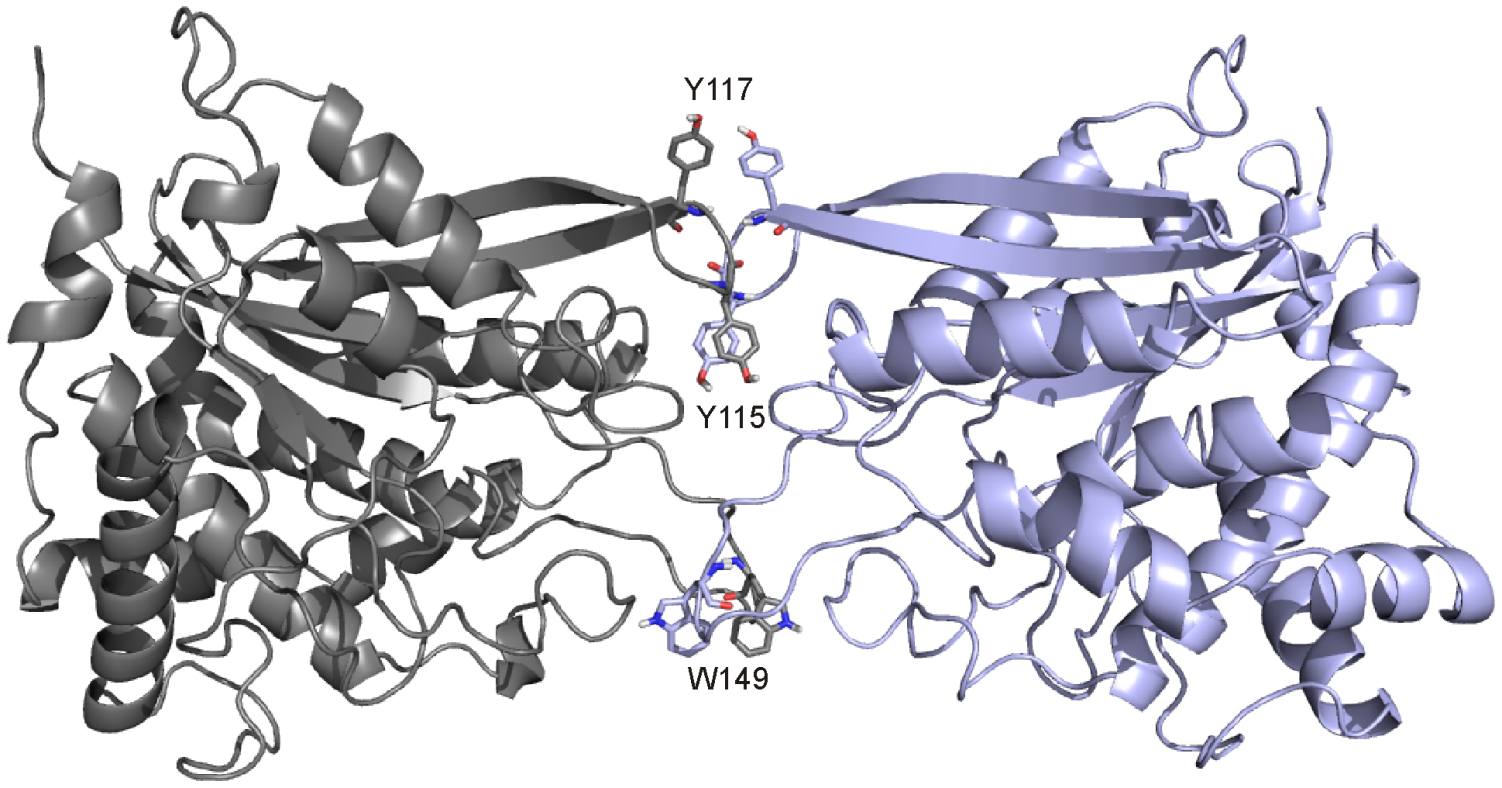
Structural model of the hQPCT homodimer. Ribbon representation of the lowest energy structure selected among 173 conformers calculated by HADDOCK. The three hQPCT residues (i.e., Y115, Y117, W149) more than 11 Å far apart from the metal ion involved in intermolecular hydrophobic contacts are shown as sticks.

The ensemble of structural models obtained for the homodimer is a cluster of 173 conformers with a RMSD of 0.7±0.4 Å from the overall lowest energy structure. Four residues with aromatic side chains are the most involved in the intermolecular hydrophobic contacts in 173 model structures (Table S1). They were Y115 and Y117 in the hydrophobic region 1, H148 and W149 in the hydrophobic region 2 (Fig. 5) and have been selected as the candidate residues for mutation into Glu/Asp. In the hydrophobic region 1 two mutations into Glu were introduced at positions 115 and 117 (Y115E and Y117E) to provide the variant called 2xmut hQPCT. The spatial proximity of the two residues in the cDNA sequence has the advantage of introducing both mutations in a single mutagenesis round. Additionally, in hydrophobic region 2, W149 was mutated into Asp in the variant called 6xmut (see below). H148 was left unchanged to avoid destabilization of the zinc binding site, as described in Materials and Methods.

### Production of the 2xmut and 6xmut hQPCT

**Figure 6:**
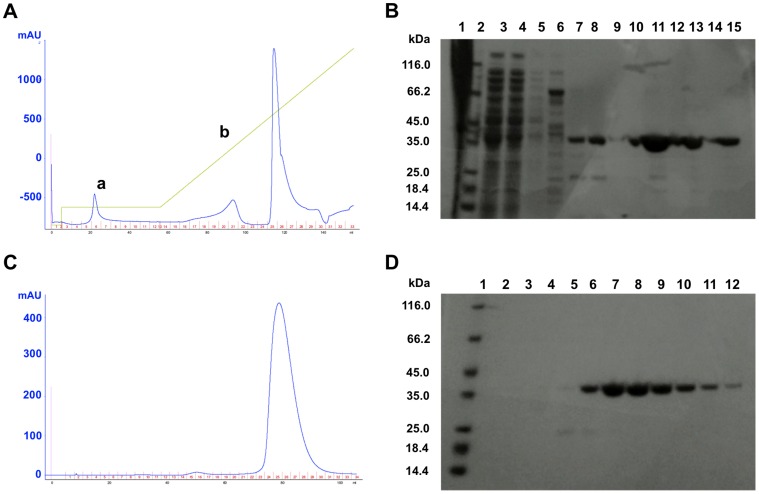
Purification of 2xmut hQPCT. **A,** Ni-NTA affinity purification-imidazole gradient (green line: a, 50 mM; b, 50–500 mM) in FPLC Akta (GE Healthcare). Blue line: UV measure (mAU). **B,** SDS-PAGE gel of imidazole gradient fractions, lane 1: insoluble fraction, lane 2: protein marker, lane 3: total fraction, lane 4: flow-through, lane 5: wash unbound, lanes 7–15: fractions 50–500 mM imidazole. **C,** Size Exclusion in HiLoad 16/60 Superdex 75 column in FPLC Akta (GE Healthcare). Blue line: UV measure (mAU). **D,** SDS-PAGE, lane 1: protein marker, lanes 6–12: monomeric 2xmut hQPCT.

For the 2xmut hQPCT production, we used the same conditions as for the wild-type hQPCT. We first performed a mini-scale expression test in 100 ml SB to check protein expression and solubility (data not shown). Once confirmed, the protein expression was carried out in 1 l M9 medium enriched with ^15^N and ^13^C and induced with 0.2 mM IPTG. At this point, the growth temperature was shifted to 17°C for 48 hours. After cell collection and lysis, the sample was loaded on a Ni-affinity HisTrap column and the protein was eluted as previously described (Fig. 6A).

Opposite to what observed in wild-type hQPCT purification (Fig. 2), little protein was found in inclusion bodies whereas the greatest amount of hQPCT was detected in the soluble fraction (Fig. 6B). In this case, the final yield was around 22 mg/l, similar to what was obtained in wild-type expression in rich medium, thus indicating that the mutations introduced in the sequence improved protein solubility. A second step of protein purification using a HiLoad 16/60 Superdex 75 column was performed and showed almost no protein aggregation (Fig. 6C). 2xmut hQPCT was eluted in 150 mM NaCl, 50 mM Tris buffer, pH 8 and purity was checked by SDS-PAGE (Fig. 6D).

In order to check the zinc(II) content of the 2xmut hQPCT, an atomic absorption spectroscopy analysis was performed. The results revealed that the 2xmut hQPCT protein obtained from our expression has a degree of metalation <40%. To ensure full charging with Zn, the enzyme was reconstituted with the metal *in vitro* by dialysis as described in Materials and Methods. The 2xmut hQPCT partially metalated and fully metalated presented the same secondary structure element content, as shown by CD measurements, as well as tertiary structure, as demonstrated by the HSQC spectra, both showing superimposable profiles (data not shown). In addition, the CD spectrum acquired on the 2xmut hQPCT showed the same profile of the wild type, thus indicating that the introduced mutations do not affect the secondary structure of the protein.

Afterwards, the formation of the intra-chain disulfide bond of the double mutant was investigated. hQPCT contains two cysteine residues at amino acid positions 139 and 164 (Fig. 1). Disulfide bonds were reported to be present in almost 50% of the protein expressed in *E. coli* [6]. The intra-chain disulfide bond status for the 2xmut hQPCT was checked by the modification of the two free thiol groups with 4-acetamido-4′-maleimidylstilbene-2,2′-disulfonic acid (AMS) (see Materials and Methods). The SDS-PAGE analysis performed on the protein obtained from our expression showed a disulfide bond content <50%. Attempts to oxidize the 2xmut hQPCT by adding a 1000-fold excess of ferricyanide to the protein solution or treating the enzyme with a redox couple (2-Mercaptoethanol: 2-Hydroxyethyl disulfide at molar ratios of 1∶2 and 1∶10) by a series of dialysis failed to yield an appreciable increase in the amount of disulfide bonds. Despite the low content of disulfide bonds of the 2xmut hQPCT, activity tests performed on the fully metalated protein showed good enzymatic activity. The enzymatic activity was tested with a fluorometric assay based on the conversion of the H-Gln-AMC substrate [26] and compared with that of a human wild type recombinant QPCT obtained commercially. The 2xmut hQPCT showed a significant activity already at 1 nM (Fig. S1) that was comparable with the activity of the same concentration of the commercially available wild type recombinant human QPCT. To assess if the mutation could affect the interaction with a known inhibitor in a functional mode, experiments were repeated in the presence of the PBD150 QPCT inhibitor [27]. The concentration response curves obtained for the two enzymes (Fig. S2) showed equal IC_50_ values, thus indicating that the mutations introduced were not affecting the inhibitor-enzyme affinity.

Maintained activity also for a protein with only partial disulphide bond formation suggests that this structural element has mainly a stabilizing role. The zinc active site of hQPCT is hosted in a solvent exposed area formed by several loops connecting different secondary structure elements, namely α3 with α4, β3 with α5, β4 with α7, β5 with α8, α8 with α9, β6 with α10 (Fig. 1) [4]. The two Cys residues forming the bridge are located at the N-term of α5-helix and at C-term of β3-strand, at the basis of the large cavity hosting the zinc(II) ions. A catalytic site formed only by loops is a quite peculiar feature in zinc-dependent exopeptidases (http://scop.mrc-lmb.cam.ac.uk/scop/): lack of any rigid structural elements makes it quite flexible and most probably able to tolerate the small structural differences that may be caused by differences in the oxidation state of the two cysteines. Although the disulphide bridge may have a role for the overall structural stability, the secondary structure of the protein, determined via CD, is not influenced by the oxidation state of the disulphide bridge. This observation is consistent with the fact that the two Cys residues are located at the end of α-helix and of a β-strand. Additionally, the pattern of resonances in our HSQC spectra (see below) is consistent with a protein with defined tertiary structure. On the other hand, our data don’t allow us to rule out that the absence of the bond may influence the stability of the protein towards unfolding.

Similar results were obtained with the 6xmut hQPCT: protein expression was carried out in 1 l SB medium following the same conditions as for the 2xmut or wild-type hQPCT. After the first step of purification (Affinity column), the yield of protein was around 150 mg/l and little protein was found in inclusion bodies (Fig. S3A). Further purification steps through size exclusion columns showed that most of the purified protein was found in the monomeric form (Fig. S3B).

In order to check the zinc(II) content of the 6xmut hQPCT, atomic absorption spectroscopy analysis was performed. The results revealed that the protein was 95% metallated.

6xmut hQPCT resulting from purification process was used to check enzymatic activity and was found to have significant activity, comparable to the commercially available wild type recombinant human protein (Fig. S4).

Finally, the 6xmut hQPCT presents the same secondary structure elements of the wild type and of the 2xmut, as proved from the comparison of the CD spectra (data not shown), as well as a very similar HSQC spectrum (see below).

### NMR Spectra

The maximum solubility accessible in stable solution of wild type hQPCT was 30 µM. The ^15^N HSQC acquired at this concentration is shown in Figure 3A. Despite the high number of scans used, the signal intensity is very low, due to the extremely low concentration of a non-deuterated sample of a protein of this size. Nevertheless, the HSQC spectrum shows a good chemical shift dispersion (6.0–10.5 ppm) indicating that the protein is folded, as also confirmed by the well resolved proton resonances of methyl groups in the upfield region of the ^1^H 1D NMR spectrum (Fig. S5). Solubility tests performed on the 2xmut hQPCT sample proved that the maximum achievable protein concentration was 200 µM. The ^15^N HSQC recorded on the 90 µM ^15^N,^13^C 2xmut hQPCT (Fig. 3B) contains a larger number of peaks with respect to wild type hQPCT, that span the 6–12 ppm range. The larger number of detectable resonances is attributable to the higher protein concentration and consequent better signal- to-noise ratio. The peak line width is consistent with the large protein size and signals are well dispersed over the observed chemical shift range, as expected for a structured protein. A central region crowded of overlapped peaks (7–9 ppm) is a consequence of the structure of hQPCT, where the coil and loop regions represent 42% of the overall secondary structure, and with the presence of a large (36%) α-helical content [4]. The HSQC spectra of the partially and fully metalated 2xmut hQPCT are superimposable, thus indicating that the metal binding do not significantly affect the structure of the zinc enzyme as expected on the basis of the X-ray structure that shows an exposed metal binding site [4].

The 6xmut hQPCT sample designed for purposes outside of this work is more soluble with respect to the wild type hQPCT but slightly less soluble in comparison to the 2xmut hQPCT with a maximum achievable concentration of 150 µM. Indeed, besides the Y115E, Y117E and W149D mutations, expected to provide an increase in protein solubility, the three additional mutations S119A, S121A and N150P play an opposite role. Nevertheless, the fact that a variant that contains three mutations that are in principle counterproductive towards solubility is still much more stable than our single-point mutants, indicates that the solubility of hQPCT is largely governed by localized effects related to the presence of aromatic residues in the large hydrophobic patches rather than by a generic surface hydrophobicity. The ^15^N HSQC performed on the 70 µM ^15^N,^13^C 6xmut hQPCT is reported in Figure 3C. NMR was also used to demonstrate the binding of the ligand PBD150, a known inhibitor of wild type hQPCT [13] that we have found to inhibit also the activity of the 2xmut (see above). A titration of the 2xmut ^15^N labeled protein with increasing amounts of this inhibitor was performed. Fast and intermediate exchange regimes on the NMR chemical shift time scale between free and bound forms of the protein were observed for different signals. Indeed, binding of PBD150 to hQPCT resulted in the chemical shift variation as well as in the disappearance of a number of resonances in the ^1^H–^15^N HSQC spectrum of the 2xmut enzyme.

### Prospects

NMR characterization of proteins relies on isotopically enriched samples: ^15^N,^13^C-labeling is generally used for protein assignment via triple resonance experiments while at least partial ^2^H-enrichment is required for proteins with molecular mass above 30 kDa. The requirement of isotopically-enriched and concentrated (tens of mM or above) samples implies that NMR studies are generally conducted on recombinant proteins, most often expressed in *E. coli*. Lack of post-translational modifications and the presence of tags for cloning or purification purposes reflect on protein solubility, inducing formation of intermolecular adducts ranging from large insoluble aggregates to partially populated small oligomers [28], [29]. Here we proposed the use of surface analysis and docking programs to derive hypothesis-driven models for the intermolecular interactions on the basis of the oligomerization for the efficient and rational design of more soluble mutants.

The attainment of relatively soluble variants of hQPCT opens new routes for development of novel inhibitors of the Glutaminyl Cyclase function. On one side, the ability to monitor binding of PBD150 at the catalytic site via resolved resonances in ^1^H-^15^N HSQC spectra anticipates the successful use of this technique for NMR screening studies on hQPCT. On the other, the ability to maintain the protein in a soluble, monodispersed state facilitates crystal growth for X-ray crystallography.

## Materials and Methods

### Cloning and Mutagenesis

**Figure 7:**
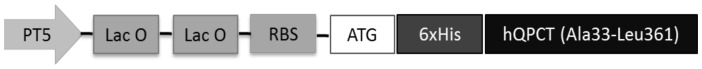
pQE80 plasmid encoding hQPCT sequence. *PT5*, promotor T5; *Lac O*, lac operator element; *RBS*, ribosomal binding site; *ATG*, Methionine codon; *6xHis*, His_6_ tag coding sequence; *hQPCT*, human QPCT sequence from Ala33 to Leu361.

The cDNA sequence encoding the human QPCT (Ala33-Leu361) was amplified by PCR using sequence-specific oligos flanked by *BglII* (forward AACAGATCTGCCTCAGCCTGGCCAGAG) and *HindIII* (reverse CGAAGCTTTTACAAATGAAGATATTCCAACAC) restriction sites. The resulting product was cloned into the pQE80L vector (Qiagen) linearized with *BamHI* and *HindIII* restriction enzymes (Fig. 7).

For the production of the double-mutated hQPCT (Y115E-Y117E), called 2xmut hQPCT, a set of oligos containing both the desired mutations were used: Y115E-Y117E forward 5′ CTTGAGTCAGACACCCGAAGGGGAACGGTCTTTCTCAAATATC 3′, Y115E-Y117E reverse 5′ GATATTTGAGAAAGACCGTTCCCCTTCGGGTGTCTGACTCAAG 3′. For the production of the 6xmut hQPCT two different set of oligos were used: first set Y115E-Y117E-S119A-S121A forward 5′ CTTGAGTCAGACACCCGAAGGGGAACGGGCTTTCGCAAATATC 3′, reverse 5′ GATATTTGCGAAAGCCCGTTCCCCTTCGGGTGTCTGACTCAAG 3′; second set W149D-N150P forward 5′ CCAAGTATTTTTCCCACGACCCCAACAGAGTGTTTGTAGG 3′, W149D-N150P reverse 5′ CCTACAAACACTCTGTTGGGGTCGTGGGAAAAATACTTGG 3′. The pQE80L-hQPCT (Ala33-Leu361) vector was used as DNA template and reactions were set up as indicated in the QuickChange Site-directed Mutagenesis Kit (Agilent) manufacturer’s protocol.

### Expression and Purification

For protein expression, BL21 (DE3) *E. coli* cells were transformed with pQE80L vectors either encoding hQPCT or 6xmut hQPCT or 2xmut. Cells were grown at 37°C in rich (SB) or minimal medium (M9) till OD_600_ reached 0.6–0.8 and then, protein expression was induced with 0.2 mM IPTG. Cells were then further incubated at 17°C for 48 hours. After two days, cells were collected (4,000 *g* 15 minutes) and resuspended in an appropriate volume of lysis buffer (150 mM NaCl, 50 mM Tris, pH 8, 20 mM imidazole). Lysis was carried out by sonication. The lysate was then clarified by centrifugation at 30,000 rpm for 30 minutes and the final supernatant was filtered through a 0.2 µm filter. Pellet was stored for inclusion bodies analysis.

A two-step purification protocol was used. First, the supernatant was loaded into a Ni-affinity HisTrap FF 5 ml column (GE Healthcare) and bounded proteins were eluted with a gradient of imidazole (50–500 mM). Next, fractions containing the his-tagged hQPCT were pooled and loaded into a size exclusion chromatography column (HiLoad 16/60 Superdex 75, Pharmacia). Protein fractions were recovered in 150 mM NaCl, 50 mM Tris, pH 8 buffer.

### Metalation

The 2xmut hQPCT was loaded with zinc by overnight dialysis at 4°C against a 150 mM NaCl, 50 mM Tris, pH 8 buffer in presence of an equimolar amount of ZnCl_2_. Unbound metals were subsequently removed by two steps of dialysis. Finally the content of zinc was measured by ICP-MS showing a 200% of metal content.

### Circular Dichroism Spectroscopy Analysis

CD spectra to assess the protein secondary structure were acquired with a Jasco J-810 spectropolarimeter at 25°C using a 0.1 cm path length quartz cuvette. Buffer exchange of hQPCT with 50 mM potassium phosphate, 150 mM sodium fluoride, pH 7 buffer was performed. The mean of 10 scans between 190 and 250 nm wavelength was calculated by subtraction of the buffer spectrum.

### Disulfide-bond Analysis

To verify the disulfide-bond status AMS test was performed. The reaction of AMS with two free thiol groups increases hQPCT molecular weight of 980 Da. AMS test was carried out in oxygen-free conditions to avoid the oxidation of free thiol groups. 100 µM hQPCT was precipitated with 10% trichloroacetic acid (TCA) and washed with acetone. After centrifugation, pellet was resuspended in 100 mM Tris, 2% SDS pH 7 buffer and incubated with and without 0.1 M AMS at 37°C for 1 hour. Finally, samples were subjected to SDS-PAGE analysis.

### Sample Preparation for Mass Spectrometry and NMR Analysis

For mass spectrometry, the sample was concentrated to 55–75 µM using a 10 kDa cut-off centricon membrane (Millipore). Buffer exchange to 100 mM NH_4_Cl pH 8 was carried out using a PD-10 column. Protein demetalation (apo-hQPCT) was carried out by a series of dialysis with 150 mM NaCl, 50 mM Tris, pH 8, 10 mM EDTA.

For NMR analysis, the protein was concentrated to 100–150 µM in 150 mM NaCl, 50 mM Tris, pH 8.

### ICP-MS Analysis

The atomic absorption spectroscopy analysis was performed using a Spectro Ciros charge-coupled device inductively coupled plasma optical emission spectrometer (Spectro Analytical Instruments) in combination with a Lichte nebulizer and a peristaltic pump for sample introduction. Concentrated stocks of proteins were diluted to a concentration of 3–5 µM using 10% nitric acid distilled water. The inductively coupled plasma was programmed to detect three wavelengths for the Zn (202, 206, 213 nm) and each measurement being repeated three times. The standardization curve was made using standard solutions in the range 0–10 µM Zn in milliQ water.

### Activity Measurements

Glutaminyl cyclase activity was estimated fluorometrically by a coupled assay using pyroglutamyl aminopeptidase (from *Bacillus amyloliquefaciens*, expressed in *E. coli*, purchased from QIAGEN) as auxiliary enzyme and H-Gln-AMC as substrate (Bachem AG, Switzerland), adapting an assay described previously [26].

The assay was adapted to 384 well plate format for minimal reagent consumption and was conducted in 50 mM Tris HCl pH 8.0 in 384 well black non binding surface plates (Corning Costar) in a final 50 µl volume.

Reaction mixture contained 50 µM H-Gln-AMC (7-amino-4-methylcoumaride), the mutated hQPCT enzyme at a final concentration ranging between 1 and 100 nM and 0.2 U/ml pyroglutamyl aminopeptidase. As a positive control a commercial wild type human recombinant QPCT expressed in HEK293 (OriGene, MD, USA; protein purity >80%) was used at a standard concentration of 1 nM.

For compound inhibition tests the assay was conducted in presence of 1% DMSO and PBD150 [27] at a concentration ranging from 50 µM to 0.02 µM.

The product development was followed at 25°C by repeated kinetic fluorescence readings on a TECAN Safire2 plate reader with excitation/emission wavelengths of 380/465 nm. Fluorescence was read every 2 minutes for 40 minutes and enzyme activity was calculated as RFU/min from the linear part of the product development curve.

### NMR Experiments

Monodimensional ^1^H and ^1^H-^15^N HSQC NMR experiments were acquired on the wild type ^15^N hQPCT and on the multiple mutants of hQPCT at 298 K using Bruker Advance spectrometers operating at proton frequencies of 700 MHz and 950 MHz; both spectrometers were equipped with cryoprobes. The monodimensional ^1^H spectra were acquired on the wild type ^1^H hQPCT with 16384 points corresponding to an acquisition time of 45.5 ms.

The 2D ^1^H-^15^N HSQC was acquired on the wild type ^15^N hQPCT with 1024 points in the direct dimension and 128 in the indirect dimension corresponding to acquisition times of 45.6 and 22.5 ms. 512 transients were acquired per t1 increment. The 2D ^1^H-^15^N HSQC on the ^15^N hQPCT 2xmut was recorded with 1024 points in the direct dimension and 128 in the indirect dimension corresponding to acquisition times of 27.1 and 16.6 ms. 64 transients were acquired per t1 increment. The 2D ^1^H-^15^N HSQC on the ^15^N hQPCT 6xmut was recorded with 1024 points in the direct dimension and 200 in the indirect dimension corresponding to acquisition times of 27.1 and 26.0 ms. 256 transients were acquired per t1 increment. For these experiments the recycle delay was set to 1 s. Spectra were processed with the program TopSpin 2.0 (Bruker).

### Protein Surface Analysis

The software PyMOL (The PyMOL Molecular Graphics System, Version 1.5.0.4 Schrödinger, LLC) was used to generate the electrostatic representation of the protein surface using the command “generate vacuum electrostatic” which uses the Amber99 force field charge distribution. The evaluation of residue accessibility to the solvent for each amino acid of hQPCT was performed with the program Naccess [24]. The coordinates of the crystal structure of human glutaminyl cyclase (PDB id 2AFM) were used as input. The aromatic amino acids on three of the loops forming the crown-like structure around the zinc and on the loop connecting β1 and β2 (Fig. 1) and more than 11 Å far apart from the metal ion and having an absolute residue accessibility from Naccess >100 were defined as active residues in HADDOCK calculation (i.e., 115, 117 on the hydrophobic region 1 and 148, 149 on the hydrophobic region 2). The set of active residues was completed by hydrophobic nearby amino acids with an absolute residue accessibility >50 (i.e. 145, 205 on the hydrophobic region 1 and 147, 153 on the hydrophobic region 2).

Two-hundred structural models of the hQPCT homodimers calculated with the program HADDOCK [30], [31] clustered in only two clusters. The first one contains 173 structures and was used as our reference model. The most frequent intermolecular contacts came from the residues on the two hydrophobic regions, as summarized in Table S1. In particular Trp149 and His148 on the hydrophobic region 2 together with Tyr115 and Tyr117 on the hydrophobic region 1 are involved in the highest number of non-bonded contacts. Our mutation strategy was based on substituting residues giving rise to the highest number of non-bonded contacts. In the 2xmut hQPCT Tyr115 and Tyr117 on the hydrophobic region 1 were changed into Glu. In the 6xmut hQPCT, also Trp149 was mutated into Asp, while maintaining unaltered His148, to avoid possible electrostatic destabilization of its loop, which carries the Zn ligand Asp159. Indeed, the simultaneous introduction of two negatively charged amino acids in place of Trp149 and His148 may induce a conformational change of the loop, thus altering the coordination sphere and possibly affecting the enzymatic activity.

## Supporting Information

Figure S1 **Glutaminyl cyclase activity test for 2xmut hQPCT.** The graph shows the progress curves of AMC fluorescence development with different amounts of metalated 2xmut hQPCT or commercially available wild type recombinant human QPCT. The assay was conducted in 50 mM Tris HCl pH 8.0 at 25°C with 50 µM H-Gln-AMC, 0.2 U/ml pyroglutamyl aminopeptidase with hQPCT at the indicated concentrations. The final assay volume was 50 µl in 384 well plate.
(TIF)

Figure S2 **Enzyme inhibition test for 2xmut hQPCT.** Concentration response curve for the PBD150 reference inhibitor tested on the metalated 2xmut QPCT at a concentration of 2 nM (left panel) and on the wild type recombinant human QPCT at 1 nM (right panel) with the described fluorometric assay. Data were normalized to the relative negative control wells, set to 100%, that contained 1% DMSO in place of the compound. The calculated IC_50_ values indicate similar compound potency on the two enzymes.
(TIF)

Figure S3 **Purification of 6xmut hQPCT.** **A,** left, SDS-PAGE of fractions obtained after affinity purification (imidazole gradient). Lane 1: insoluble fraction, lane 2: marker, lane 3: total fraction, lane 4: flow-through fraction, lane 5: wash unbound fraction, lanes 6–10∶6xmut hQPCT fractions (50–500 mM imidazole gradient); right, SDS-PAGE of diluted fractions. Lane 1: marker, lane 2∶1/10 dilution of fraction of lane 8 in the left panel, lane 3∶1/10 dilution of fraction of lane 9 in the left panel; **B,** SDS-PAGE of fractions after size exclusion chromatography in HiLoad 16/60 Superdex 75 column, lane 1: marker, lanes 6–13: monomeric 6xmut hQPCT.
(TIF)

Figure S4 **Glutaminyl cyclase activity test for 6xmut hQPCT.** AMC fluorescence development with different amounts of metalated 6xmut hQPCT or wild type recombinant human QPCT commercially available. The assay was conducted in 384 well plate in 50 mM Tris HCl pH 8.0 at 25°C with 50 µM H-Gln-AMC, 0.2 U/ml pyroglutamyl aminopeptidase and hQPCT at the indicated concentrations.
(TIF)

Figure S5 **^1^H 1D NMR spectrum of 2xmut hQPCT.** Resolved signals in the upfield region of the spectrum are indicative of a folded protein.
(TIF)

Table S1 **Intermolecular contacts statistics over the 173 structures of cluster 1.** Non-bonded contacts calculated over all the 173 model structures of cluster 1 obtained by HADDOCK. Only the intermolecular contacts involving residues located >11 Å far apart from the metal ion and having a repetition frequency >20 are listed. The repetition frequency is the number of times that each contact appears in either of the two subunits of the dimeric complex, divided by 2.
(DOCX)
