# Supplementary figures and images for: Soluble Variants of Human Recombinant Glutaminyl Cyclase
Source: PLoS One. 2013 Aug 15;8(8):e71657. doi: 10.1371/journal.pone.0071657 (PMC3744504; doi:10.1371/journal.pone.0071657)

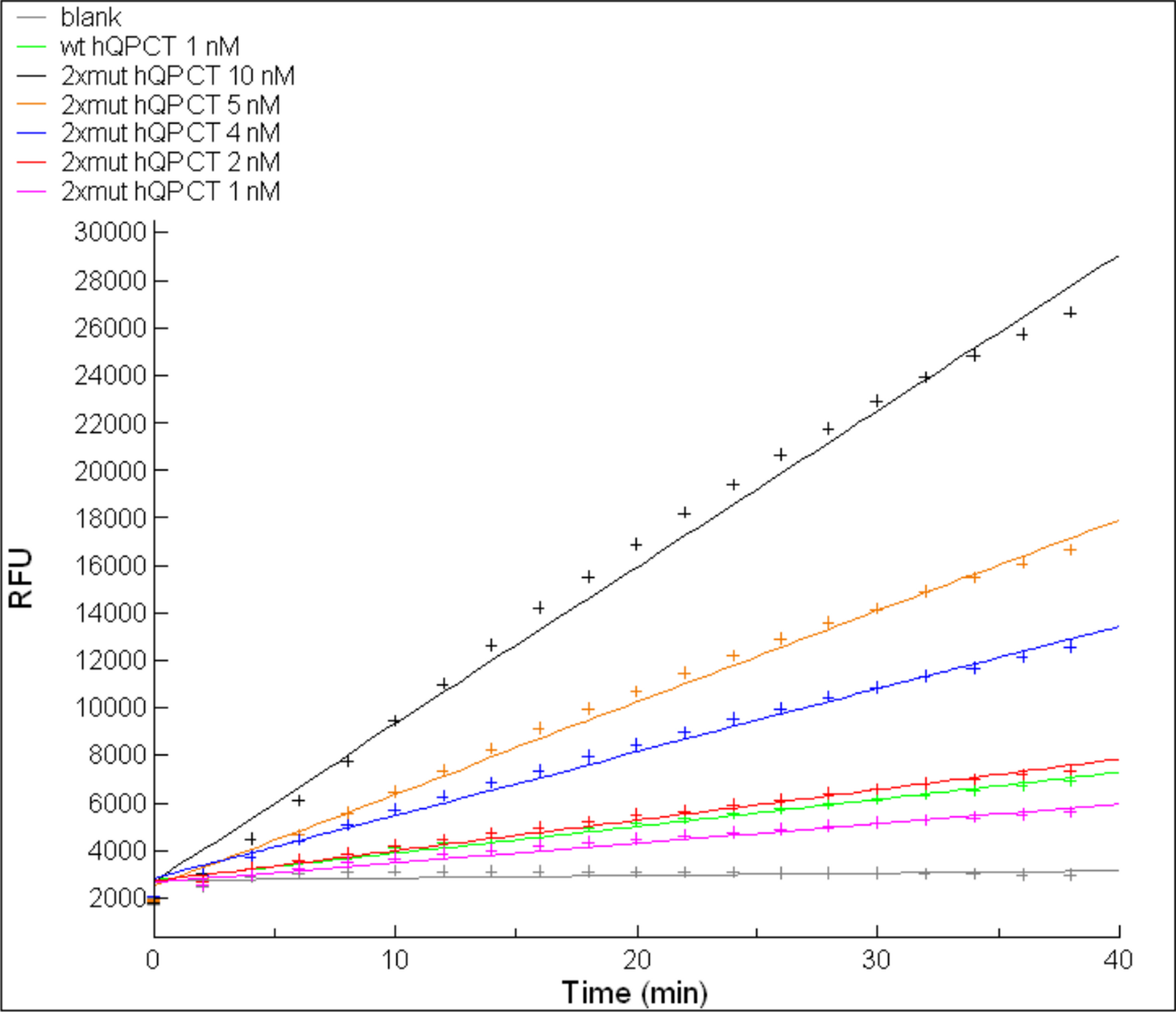

Supplement: Figure S1 — Glutaminyl cyclase activity test for 2xmut hQPCT. The graph shows the progress curves of AMC fluorescence development with different amounts of metalated 2xmut hQPCT or commercially available wild type recombinant human QPCT. The assay was conducted in 50 mM Tris HCl pH 8.0 at 25°C with 50 µM H-Gln-AMC, 0.2 U/ml pyroglutamyl aminopeptidase with hQPCT at the indicated concentrations. The final assay volume was 50 µl in 384 well plate. (TIF) [file pone.0071657.s001.tif]

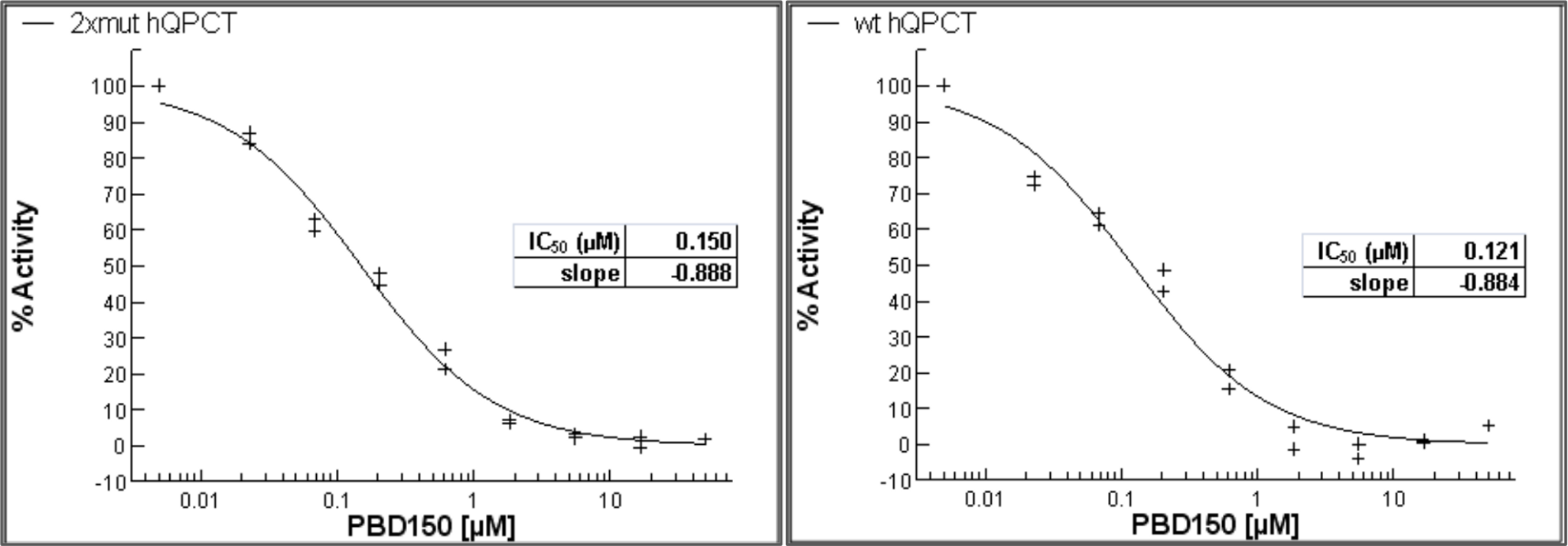

Supplement: Figure S2 — Enzyme inhibition test for 2xmut hQPCT. Concentration response curve for the PBD150 reference inhibitor tested on the metalated 2xmut QPCT at a concentration of 2 nM (left panel) and on the wild type recombinant human QPCT at 1 nM (right panel) with the described fluorometric assay. Data were normalized to the relative negative control wells, set to 100%, that contained 1% DMSO in place of the compound. The calculated IC50 values indicate similar compound potency on the two enzymes. (TIF) [file pone.0071657.s002.tif]

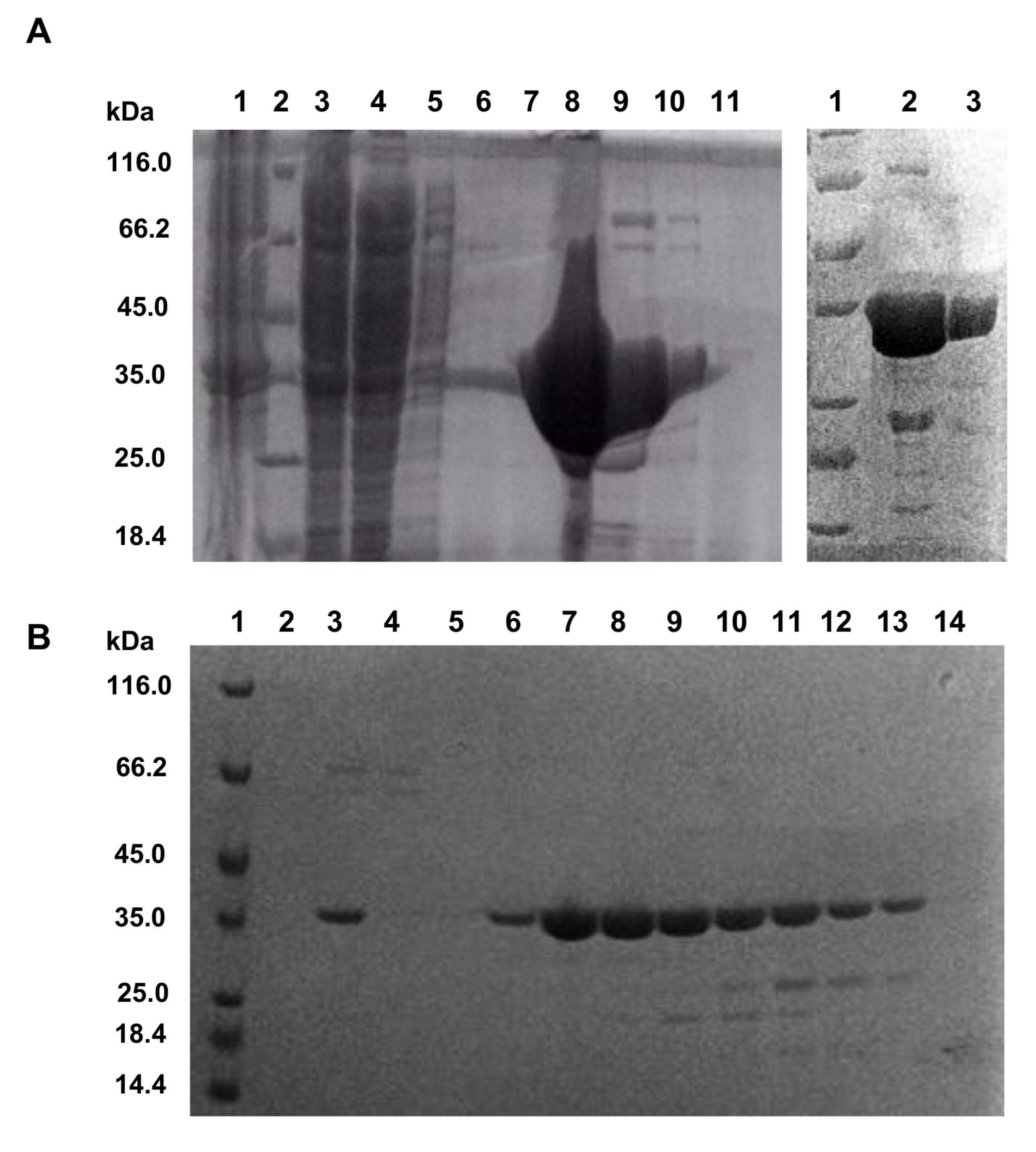

Supplement: Figure S3 — Purification of 6xmut hQPCT. A, left, SDS-PAGE of fractions obtained after affinity purification (imidazole gradient). Lane 1: insoluble fraction, lane 2: marker, lane 3: total fraction, lane 4: flow-through fraction, lane 5: wash unbound fraction, lanes 6–10∶6xmut hQPCT fractions (50–500 mM imidazole gradient); right, SDS-PAGE of diluted fractions. Lane 1: marker, lane 2∶1/10 dilution of fraction of lane 8 in the left panel, lane 3∶1/10 dilution of fraction of lane 9 in the left panel; B, SDS-PAGE of fractions after size exclusion chromatography in HiLoad 16/60 Superdex 75 column, lane 1: marker, lanes 6–13: monomeric 6xmut hQPCT. (TIF) [file pone.0071657.s003.tif]

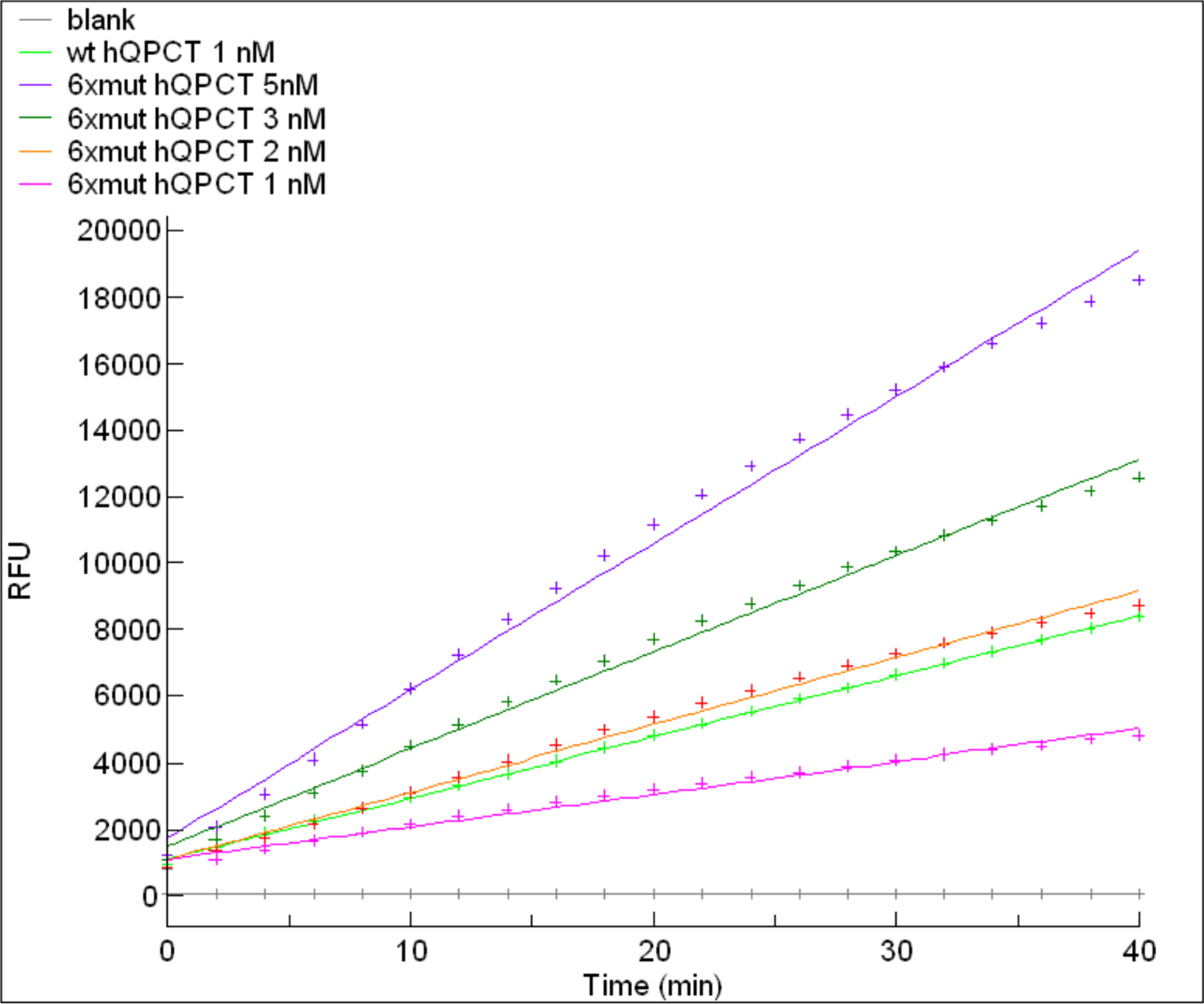

Supplement: Figure S4 — Glutaminyl cyclase activity test for 6xmut hQPCT. AMC fluorescence development with different amounts of metalated 6xmut hQPCT or wild type recombinant human QPCT commercially available. The assay was conducted in 384 well plate in 50 mM Tris HCl pH 8.0 at 25°C with 50 µM H-Gln-AMC, 0.2 U/ml pyroglutamyl aminopeptidase and hQPCT at the indicated concentrations. (TIF) [file pone.0071657.s004.tif]
