# Supplementary material for: Soluble Variants of Human Recombinant Glutaminyl Cyclase
Source: PLoS One. 2013 Aug 15;8(8):e71657. doi: 10.1371/journal.pone.0071657 (PMC3744504; doi:10.1371/journal.pone.0071657)
Supplement: Table S1 — Intermolecular contacts statistics over the 173 structures of cluster 1. Non-bonded contacts calculated over all the 173 model structures of cluster 1 obtained by HADDOCK. Only the intermolecular contacts involving residues located >11 Å far apart from the metal ion and having a repetition frequency >20 are listed. The repetition frequency is the number of times that each contact appears in either of the two subunits of the dimeric complex, divided by 2. (DOCX) [file pone.0071657.s006.docx]

**Table S1.** **Intermolecular contacts statistics over the 173 structures of cluster 1.**

| **Residues involved in intermolecular non bond contacts** | **Repetition frequency** |
| --- | --- |
| Tyr115 (Cε1, Cζ) − Tyr78 (Cδ2, Cε2, Cζ)  Tyr115 (Cβ, Cδ1, Cδ2, Cε1, Cε2, Cγ, Cζ) − Tyr115 (Cβ, Cδ1, Cδ2, Cε1, Cε2, Cγ, Cζ)  Tyr115 (C’, Cα, Cδ1, Cε1) − Arg118 (Cζ, Cγ, Cδ) | 53  144  66 |
| Tyr117 (Cβ, Cδ1, Cε1) − Tyr117 (Cδ1, Cε1, Cβ) | 24 |
| His148 (Cβ, Cδ2, Cε1) − Leu205 (C’, Cα, Cβ, Cδ1, Cδ2)  His148 (C’, Cβ, Cγ, Cδ2, Cε1) − His206 (Cβ, Cδ2, Cε1, Cγ) | 38  95 |
| Trp149 (Cβ, Cδ1, Cδ2, Cε2, Cε3, Cγ, Cζ2, Cζ3, Cη2) − Trp149 (Cβ, Cδ1, Cδ2, Cε2, Cε3, Cγ, Cζ2, Cζ3, Cη2)  Trp149 (Cδ1, Cε2, Cζ2, Cη2) − Thr331 (Cα, Cβ, Cγ2)  Trp149 (Cδ1, Cδ2, Cε2, Cζ2, Cη2, Cγ) − Met332 (Cβ, Cγ, Cε) | 171  78  41 |

Non-bonded contacts calculated over all the 173 model structures of cluster 1 obtained by HADDOCK. Only the intermolecular contacts involving residues located >11 Å far apart from the metal ion and having a repetition frequency >20 are listed. The repetition frequency is the number of times that each contact appears in either of the two subunits of the dimeric complex, divided by 2.
